# Febuxostat may decrease the incidence of COVID-19 infection among patients with gout: a retrospective cohort study

**DOI:** 10.3389/fphar.2025.1654173

**Published:** 2025-10-29

**Authors:** Weijie Wang, Shiow-Ing Wang, Yang Cheng, Xinchang Wang, Yongsheng Fan, James Cheng-Chung Wei

**Affiliations:** ^1^ State Key Clinical Specialty of Rheumatology, Department of Rheumatology, The Second Affiliated Hospital of Zhejiang Chinese Medical University, Hangzhou, China; ^2^ Center for Health Data Science, Department of Medical Research, Chung Shan Medical University Hospital, Taichung, Taiwan; ^3^ Institute of Medicine, Chung Shan Medical University, Taichung, Taiwan; ^4^ The Second Clinical Medical College of Zhejiang Chinese Medical University, Hangzhou, China; ^5^ School of Basic Medicine, Zhejiang Chinese Medical University, Hangzhou, China; ^6^ Department of Allergy, Immunology and Rheumatology, Chung Shan Medical University Hospital, Taichung, Taiwan; ^7^ Graduate Institute of Integrated Medicine, China Medical University, Taichung, Taiwan

**Keywords:** febuxostat, COVID-19, serum uric acid, collaborative network, gout

## Abstract

**Background:**

As COVID-19 infection causes a kidney proximal tubule dysfunction with urinary loss of uric acid. Hypouricemia has been found in patients with severe COVID-19 disease. However, gout is a risk factor for COVID-19 incidence and COVID-19-related death. It is not known whether urate-lowering therapy could reduce the risk of infection of COVID-19 in gout patients or not.

**Methods:**

Data from collaborative electronic health records were used in this study. A total of 663,729 patients with gout were enrolled between January 1, 2020 and December31, 2022 from 35,528,077 participants in US Collaborative Network with at least two visits. After exclusion and propensity score matching, 5,466 patients with Febuxostat and 5,466 patients with Allopurinol in the comparison group were selected. The hazard ratios (HRs) and 95% confidence intervals of COVID-19 incidence, and mechanical utilization were calculated between Febuxostat and Allopurinol groups. Subgroup analyses on sex, age, levels of serum uric acid, with vaccination group and sensitivity analyses for gout patients due to renal impairment or with tophus, different follow-up durations and considered competing risk were performed.

**Results:**

Compared to Allopurinol group, Febuxostat significantly reduced the risk of COVID-19 incidence (HR = 0.878 [0.801–0.963]) and hospitalization (HR = 0.874 [0.772–0.989]). Febuxostat appears to be more effective in male, elder, without record of COVID-19 vaccination, and gout patients with serum uric acid<10 mg/dL in reducing the risk of COVID-19 infection. In addition, Febuxostat markedly reduced the hospitalization (HR = 0.652 [0.485–0.877]) in gout patients due to renal impairment or with tophus and the risks of COVID-19 incidence (HR = 0.878 [0.801–0.963]).

**Conclusion:**

In this retrospective cohort study, Febuxostat use was associated with a lower risk of COVID-19 among patients with gout for 3 years follow-up, even with renal impairment or tophus.

## Introduction

More than 200 countries have been affected by the Coronavirus disease 2019 (COVID-19) pandemic that has caused more than 6 million confirmed deaths and 15 million estimated deaths (Wang H., 2022).

Renal abnormalities and gastrointestinal symptoms are common in COVID-19 patients which contributed a lot to the mortality (Austhof et al., 2022; Cheng et al., 2020). SARS-CoV-2 primarily targets the kidneys and gut, which are both important for uric acid excretion. Patients with severe COVID-19 disease had significantly lower serum uric acid concentrations (He et al., 2020; Werion et al., 2020), possibly because inflammation reduced renal tubular uric acid net reabsorption, according to recent studies. However, the retrospective study from Leishenshan Hospital in China demonstrated that the levels of uric acid≥423 μmol/L and ≤278 μmol/L were associated with increased risk of the composite outcome and mechanical ventilation (Chen et al., 2021). Additionally, hyperuricemia was found in many COVID-19 patients during favipiravir therapy (Koseki et al., 2022; Hanai et al., 2022). A population-based study in the UK Biobank cohort demonstrated that gout is a risk factor for COVID-19 incidence and COVID-19-related death (Topless et al., 2022). Several studies have provided evidence of the association between COVID-19 and gout. A large-scale cohort study indicated that gout patients still faced a higher risk of SARS-CoV-2 infection and severe disease progression even after vaccination (Xie et al., 2023). Another study from Mexico revealed that the incidence of gout flares significantly increased from 4.4% before the pandemic to 36% during the pandemic, representing an approximately 8-fold rise (García-Maturano et al., 2022). Data analysis from a global multicenter cohort (including 52,000 COVID-19 patients from China, the United States, and Brazil) revealed that the gout flare rate among patients with mild COVID-19 symptoms was 9.2%, whereas it reached 28.7% in severe cases. Moreover, among severe patients, the risk of gout flare was 3.1 times higher in those who did not regularly take urate-lowering therapy compared with those who did (Jatuworapruk et al., 2023).

Infection with the SARS-CoV-2 virus can trigger a stress response in the immune system, leading to the release of a large number of cytokines and initiating an inflammatory response (Pasrija and Naime, 2021). Gout itself is an inflammatory disease, and persistent immune system stress can amplify the inflammatory response. Concurrently, this stress disrupts the balance between uric acid production and excretion, thereby increasing the risk of gout onset (Wu et al., 2022). Notably, a key cytokine, IL-17A, has been implicated in both conditions. In gout, IL-17A plays a crucial role in mediating the inflammatory response. Intriguingly, research has found that the ORF8 protein expressed by the SARS-CoV-2 virus mimics the function of human IL-17A. It can bind to the IL-17 receptor A, even inducing a more intense inflammatory reaction than human IL-17A (Lin et al., 2023). This suggests a potential common inflammatory pathway between COVID-19 and gout. Furthermore, patients with gout frequently have a high burden of comorbidities linked to worse COVID-19 prognosis, including chronic kidney disease, hypertension, cardiovascular disease, obesity, and diabetes mellitus (Singh and Gaffo, 2020). This comorbid profile places the gout population *a priori* at a higher risk of complications from respiratory infections.

Recent studies have found shown that febuxostat, a non-purine selective inhibitor of xanthine oxidase (XO), is more effective and had a better safety outcome than allopurinol in lowering the uric acid levels in patients with hyperuricemia and gout (Konishi et al., 2022; Gao et al., 2021; Dehlin et al., 2020; Tsai et al., 2023).

Despite these strong mechanistic and epidemiological premises, and the risk between COVID-19 and gout, no large-scale study has investigated whether Febuxostat could reduce the risk of COVID-19 infections in gout patients. In the present study, we aimed to provide some evidence for Febuxostat use and the risk of COVID-19 infection.

## Methods

### Study design

**FIGURE 1:**
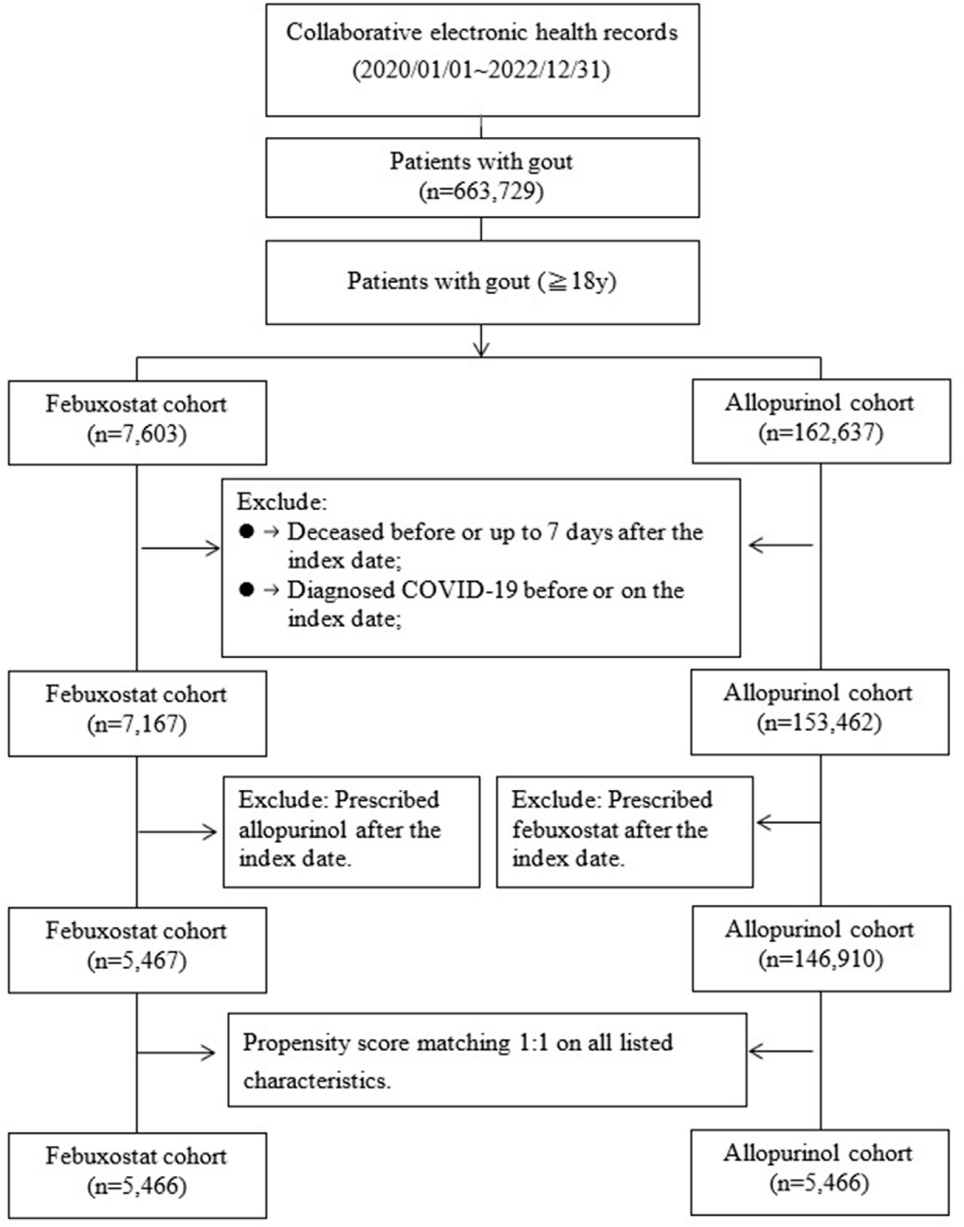
Flow chart of selection.

Propensity score matching (PSM) was performed on age, sex, race, body mass index (BMI), socioeconomic status, comorbidities, medications, medical utilization, and laboratory tests at a ratio of 1:1 in the study cohort. After PSM, 5,466 participants in the Febuxostat and 5,466 comparisons in the Allopurinol groups were selected. Figure 1 showed the selection flowchart of the cohort.

### Data sources

In the database, the largest worldwide data set for COVID-19 is stored. The study data was retrieved from 57 global healthcare organizations in the US Collaborative Network. We have previously presented in detail the data included in the database, including demographics, diagnoses, procedures, medication information, laboratory tests, genomics, and healthcare utilization (Wang W. et al., 2022). A cohort of more than 35 million participants was constructed using collaborative electronic health records.

A total of 663,729 patients with gout were enrolled between January1, 2020 and December31, 2022 from 35,528,077 participants in collaborative electronic health records with at least two visits. People aged≥18 years diagnosed with gout [ICD10 = M10, M1A] were included in the cohort (n = 630,596). In addition, two groups of participants were selected: 7,603 gout patients with Febuxostat [Anatomical Therapeutic Chemical, ATC code M04AA03, at least prescribed twice] and 162,637 gout patients with Allopurinol [ATC code M04AA01, at least prescribed twice]. The index date was the date of the first prescription during the study period. Patients diagnosed COVID-19 or deceased before or up to 7 days after the index date were excluded. Moreover, for the Febuxostat cohort, prescribed Allopurinol after the index date (switchers)and for the Allopurinol cohort, prescribed febuxostat after the index date (switchers) were also excluded. After exclusion, 5,467 patients with Febuxostat and 146,910 patients with Allopurinol in the comparison group were selected.

### Outcomes

The incidence of COVID-19 and medical utilization in patients with COVID-19 was assessed starting from 7 days after the index date to the end of follow-up (lasting 3 years). The outcomes in the study were defined as follows.

COVID-19 incidence was defined as the presence of ICD-10 codes U07.1, U07.2, J12.82 (used early in the pandemic for COVID-19 pneumonia), U09.9, or Z86.16, or a positive SARS-CoV-2 PCR test identified by CPT/LOINC codes 9088, 41458–1, 94746–5, or 94511–3. The inclusion of J12.82 ensured capture of early pandemic cases before U-codes were available, and U09.9 was included to account for cases diagnosed outside the health system but documented as post-COVID condition thereafter. The incidence of COVID-19[ICD10 = U07, U09, J12.81, J12.82, B97.29, B34.2, Z86.16 or PCR test positive: 9088, 94306–8, 41458–1, 94764–8,94511–3,94746–5. The codes for medical utilization was: hospitalization [CPT codes 1013659, 1013699, or 1013729 or inpatient encounter]; critical care services [CPT code 1013729]; and mechanical ventilation [ICD-10 procedure codes 5A1935Z, 5A1945Z, 5A1955Z, 0BH17EZ, 0BH18EZ, 0BH13EZ, ICD-9-CM code 39.65, or CPT codes 31500, 1015098, and 1022227].

### Covariates

Other covariates about demographic, lifestyles, medical utilization, comorbidities, procedure/medication and laboratory tests were defined in Supplementary Table S1.

### Statistical analysis

In order to create groups with matched baseline characteristics, we used a greedy nearest neighbor matching approach- Propensity Score Matching-with 0.1 pooled standard deviations as caliper. To evaluate the balance of baseline characteristics in populations, standardized mean differences (SMD) were used to assess the match between the two groups at a fixed 1:1 ratio on all listed characteristics. In General, SMD <0.1 is considered a small difference. In addition, an integrated Kaplan-Meier analysis was used to assess the incidence of outcomes, and a log-rank test was applied for significance testing. Moreover, a built-in Cox proportional hazard model was applied to estimate the hazard ratios between the Febuxostat and Allopurinol groups. The hazard ratio (HR) for risks of COVID-19, hospitalization, critical care service and medical ventilation was calculated for both the Febuxostat and Allopurinol groups. Statistical significance was evaluated using the 95% confidence interval (95% CI).

Four models for adjusted different variables were constructed to evaluate the risks of the outcomes. In addition, considering severe gout patients, a sensitivity analysis was performed for gout due to renal impairment (M10.3) or with tophus (M1A.XXX1). Furthermore, different follow-up duration about 7 days–90 days, 7 days–180 days, 7 days to 1year, 7 days to 2 years and 7 days to 3years were also investigated for the risks of outcomes. To make the evidence robust, the competing risks of mortality were also evaluated.

### Ethics

The use of collaborative electronic health records for the present study was approved by the Chung Shan Medical University Institutional Review Board, number CS2-21105.

## Results

### Baseline characteristics of the participants

The demographic characteristics, social economic status, lifestyles, co-morbidities, procedure of vaccination, medications, medical utilization and laboratory tests of the Febuxostat and Allopurinol groups before and after PSM are presented in Table 1. The mean age of the participants in the Febuxostat group was about 64 years after matching. Approximately 73.1% of the patients were male and the major race was White (67.9%). Most of the gout patients were complicated with hypertensive diseases (61.2% in the Febuxostat group). In addition, about 44.8% of gout patients also received corticosteroids for systemic use. Moreover, there were about 40% of the patients complicated with acute kidney failure and chronic kidney disease and the levels of creatinine in around 30% patients were more than 1.5 mg/dL. The two groups were well matched regarding social economic status, comorbidities, medications and medical utilization (SMD < 0.1).

**TABLE 1:** Baseline characteristics of study subjects (before and after PSM matching).

Characteristic	Before matching			After matching^a^		
	Febuxostat cohort (n = 5467)	Allopurinol cohort (n = 146910)	Std diff	Febuxostat cohort (n = 5466)	Allopurinol cohort (n = 5466)	Std diff
Age at index, y						
Mean ± SD	64.2 ± 13.1	65.4 ± 12.8	0.088	64.2 ± 13.1	64.1 ± 13.4	0.007
Sex, n (%)						
Male	3995 (73.1)	110973 (75.5)	0.056	3994 (73.1)	4062 (74.3)	0.028
Female	1472 (26.9)	35925 (24.5)	0.057	1472 (26.9)	1403 (25.7)	0.029
Race, n (%)						
White	3710 (67.9)	102025 (69.4)	0.034	3710 (67.9)	3747 (68.6)	0.015
Black or African American	834 (15.3)	24885 (16.9)	0.046	834 (15.3)	801 (14.7)	0.017
Asian	318 (05.8)	4542 (03.1)	0.132	317 (05.8)	337 (06.2)	0.015
American Indian or Alaska Native	13 (00.2)	348 (00.2)	<0.001	13 (00.2)	10 (00.2)	0.012
Native Hawaiian or Other Pacific	17 (00.3)	440 (00.3)	0.002	17 (00.3)	21 (00.4)	0.012
Islander						
Unknown	575 (10.5)	14670 (10.0)	0.018	575 (10.5)	551 (10.1)	0.014
Social economic status, n (%)						
Persons with potential health hazards related to socioeconomic and psychosocial circumstances	54 (01.0)	1434 (01.0)	0.001	54 (01.0)	47 (00.9)	0.013
Lifestyles, n (%)						
Tobacco use	72 (01.3)	2527 (01.7)	0.033	72 (01.3)	73 (01.3)	0.002
Nicotine dependence	241 (04.4)	8431 (05.7)	0.061	241 (04.4)	227 (04.2)	0.013
Alcohol related disease	116 (02.1)	4328 (02.9)	0.052	116 (02.1)	102 (01.9)	0.018
Medical utilization, n (%)						
Preventive medicine services	387 (07.1)	11331 (07.7)	0.024	387 (07.1)	360 (06.6)	0.020
Hospital inpatient services	632 (11.6)	16752 (11.4)	0.005	632 (11.6)	617 (11.3)	0.009
Emergency department services	1091 (20.0)	31409 (21.4)	0.035	1091 (20.0)	1029 (18.8)	0.029
Office or other outpatient services	3479 (63.6)	90446 (61.6)	0.043	3478 (63.6)	3436 (62.9)	0.016
Comorbidities, n (%)						
Hypertensive diseases	3346 (61.2)	92613 (63.0)	0.038	3345 (61.2)	3248 (59.4)	0.036
Heart failure	847 (15.5)	23024 (15.7)	0.005	847 (15.5)	783 (14.3)	0.033
Ischemic heart diseases	1016 (18.6)	29086 (19.8)	0.031	1016 (18.6)	970 (17.7)	0.022
Atrial fibrillation and flutter	773 (14.1)	21093 (14.4)	0.006	773 (14.1)	720 (13.2)	0.028
Nontraumatic intracerebral hemorrhage	10 (00.2)	390 (00.3)	0.017	10 (00.2)	13 (00.2)	0.012
Cerebral infarction	150 (02.7)	4132 (02.8)	0.004	150 (02.7)	153 (02.8)	0.003
Other peripheral vascular diseases	272 (05.0)	6823 (04.6)	0.015	272 (05.0)	245 (04.5)	0.023
Atherosclerosis	214 (03.9)	5707 (03.9)	0.002	214 (03.9)	207 (03.8)	0.007
Diabetes mellitus	1580 (28.9)	44299 (30.2)	0.027	1579 (28.9)	1494 (27.3)	0.035
Overweight and obesity	1175 (21.5)	31864 (21.7)	0.005	1174 (21.5)	1127 (20.6)	0.021
Disorders of lipoprotein metabolism	2611 (47.8)	73114 (49.8)	0.040	2610 (47.7)	2506 (45.8)	0.038
Neoplasms	1044 (19.1)	28265 (19.2)	0.004	1044 (19.1)	1034 (18.9)	0.005
Chronic lower respiratory diseases	805 (14.7)	21284 (14.5)	0.007	805 (14.7)	744 (13.6)	0.032
Anxiety, stress-related, somatoform and other nonpsychotic mental disorders	595 (10.9)	15816 (10.8)	0.004	595 (10.9)	603 (11.0)	0.005
Mood [affective] disorders	554 (10.1)	16421 (11.2)	0.034	554 (10.1)	534 (09.8)	0.012
Unspecified dementia	46 (00.8)	1469 (01.0)	0.017	46 (00.8)	37 (00.7)	0.019
Depressive episode	449 (08.2)	12779 (08.7)	0.017	449 (08.2)	432 (07.9)	0.011
Noninfective enteritis and colitis	165 (03.0)	4195 (02.9)	0.010	165 (03.0)	166 (03.0)	0.001
Diseases of liver	396 (07.2)	9809 (06.7)	0.022	395 (07.2)	355 (06.5)	0.029
Sleep disorder	1129 (20.7)	29735 (20.2)	0.010	1128 (20.6)	1042 (19.1)	0.039
Psoriasis	123 (02.2)	2245 (01.5)	0.053	123 (02.3)	129 (02.4)	0.007
Acute kidney failure and chronic kidney disease	2160 (39.5)	42760 (29.1)	0.220	2159 (39.5)	2065 (37.8)	0.035
Systemic lupus erythematosus	52 (01.0)	747 (00.5)	0.052	52 (01.0)	56 (01.0)	0.007
Dermato/polymyositis	10 (00.2)	119 (00.1)	0.028	10 (00.2)	10 (00.2)	<0.001
Disorders of bone density and structure	408 (07.5)	8922 (06.1)	0.055	408 (07.5)	377 (06.9)	0.022
Alzheimer’s disease	14 (00.3)	582 (00.4)	0.025	14 (00.3)	14 (00.3)	<0.001
Conjunctivitis	62 (01.1)	1318 (00.9)	0.024	62 (01.1)	67 (01.2)	0.008
Procedure, n (%)						
COVID-19 vaccination (BNT)^b^	116 (02.1)	2266 (01.5)	0.043	116 (02.1)	109 (02.0)	0.009
COVID-19 vaccination (Moderna)^c^	23 (00.4)	628 (00.4)	0.001	23 (00.4)	24 (00.4)	0.003
COVID-19 vaccination (Janssen)^d^	10 (00.2)	37 (00.0)	0.049	10 (00.2)	10 (00.2)	<0.001
Medication, n (%)						
NSAIDs	1309 (23.9)	36995 (25.2)	0.029	1308 (23.9)	1317 (24.1)	0.004
Corticosteroids for systemic use	2452 (44.9)	54749 (37.3)	0.155	2451 (44.8)	2489 (45.5)	0.014
Other anti-gout						
Colchicine	1166 (21.3)	20204 (13.8)	0.200	1165 (21.3)	1173 (21.5)	0.004
Probenecid	74 (01.4)	568 (00.4)	0.104	73 (01.3)	76 (01.4)	0.005
Pegloticase	12 (00.2)	34 (00.0)	0.056	12 (00.2)	10 (00.2)	0.008
Lesinurad	10 (00.2)	16 (00.0)	0.055	10 (00.2)	10 (00.2)	<0.001
Sulfinpyrazone	0 (00.0)	0 (00.0)	NA	0 (00.0)	0 (00.0)	NA
Diuretics	2029 (37.1)	53204 (36.2)	0.019	2028 (37.1)	1942 (35.5)	0.033
Ethambutol	0 (00.0)	36 (00.0)	0.022	0 (00.0)	10 (00.2)	0.061
Pyrazinamide	0 (00.0)	10 (00.0)	0.012	0 (00.0)	0 (00.0)	NA
Laboratory, n (%)						
Creatinine, ≥1.5 mg/dL	1776 (32.5)	32112 (21.9)	0.241	1775 (32.5)	1680 (30.7)	0.037
C reactive protein, ≥3.0 mg/L	500 (09.1)	11118 (07.6)	0.057	500 (09.1)	503 (09.2)	0.002
Body mass index, ≥30 kg/m^2^	1185 (21.7)	31029 (21.1)	0.014	1185 (21.7)	1140 (20.9)	0.020
Urate, ≥10 mg/dL	421 (07.7)	6751 (04.6)	0.130	421 (07.7)	401 (07.3)	0.014

^a^ Propensity score matching was performed on all listed characteristics.

^b^ CPT, code 91300.

^c^ CPT, code 91301.

^d^ CPT, code 91303.

Bold font represents a standardized mean difference >0.1.

If the patient is less or equal to 10, results show the count as 10.

SD, standard deviation; Std diff., standardized mean difference; COVID-19, Coronavirus Disease-2019; BNT, BioNTech; NSAIDs, Anti-inflammatory and anti-rheumatic products, non-steroids; NA, not applicable.

### Incidence of COVID-19 and medical utilization in the two groups

**FIGURE 2:**
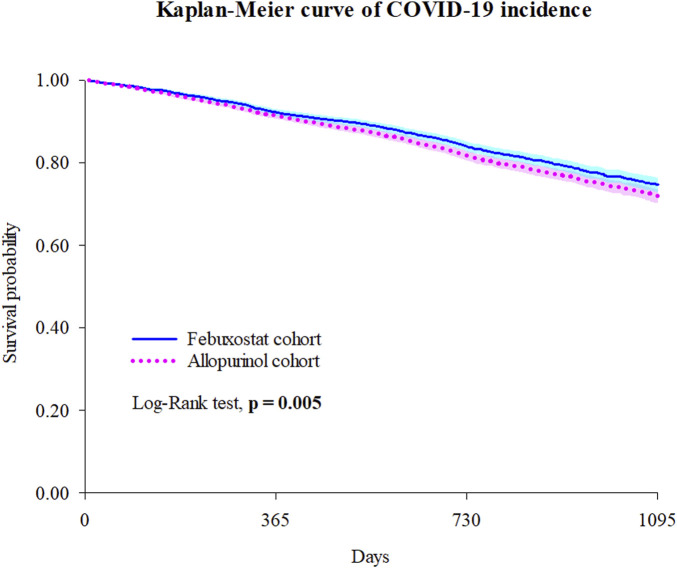
Kaplan-Meier curve of COVID-19 incidence. Bold font indicates statistical significance.

The risks of COVID-19 incidence and medical utilization in the Febuxostat and Allopurinol groups were assessed (Table 2). The HR for COVID-19 infection comparing febuxostat vs. allopurinol was 0.878 (95% CI 0.801–0.963), with an E-value of 1.54 for the point estimate and 1.24 for the upper CI limit. Compared to Allopurinol group, Febuxostat significantly reduced the risk of COVID-19 incidence (HR = 0.878 [0.801–0.963]) and hospitalization (HR = 0.874 [0.772–0.989]). The Kaplan-Meier curve of incidence of the COVID-19 outcomes also indicated a difference of probability between the two groups in Figure 2 (Log–rank test, *P* = 0.005). Even adjusted different variables, we got similar results (Supplementary Table S2).

**TABLE 2:** Risk of outcomes between Febuxostat group and Allopurinol group.

Outcomes	Patients with outcome		Adjusted^a^ hazard ratio (95% CI)	E-value for point estimate (E-value for the CI)
	Febuxostat cohort (n = 5466)	Allopurinol cohort (n = 5466)		
Disease incidence				
COVID-19 incidence	850	978	**0.878 (0.801–0.963)**	1.54 (1.24)
Medical utilization				
Hospitalization	468	532	**0.874 (0.772–0.989)**	1.55 (1.12)
Critical care service	306	351	0.880 (0.755–1.025)	1.53 (1.00)
Mechanical ventilation	169	178	0.965 (0.782–1.192)	1.23 (1.00)

^a^ Propensity score matching was performed on all listed characteristics.

* Proportionality <0.

CI, confidence interval; COVID-19, Coronavirus Disease-2019.

If the patient is less or equal to 10, results show the count as 10.

Bold values which presented the statistical significance.

### Subgroup analyses

**FIGURE 3:**
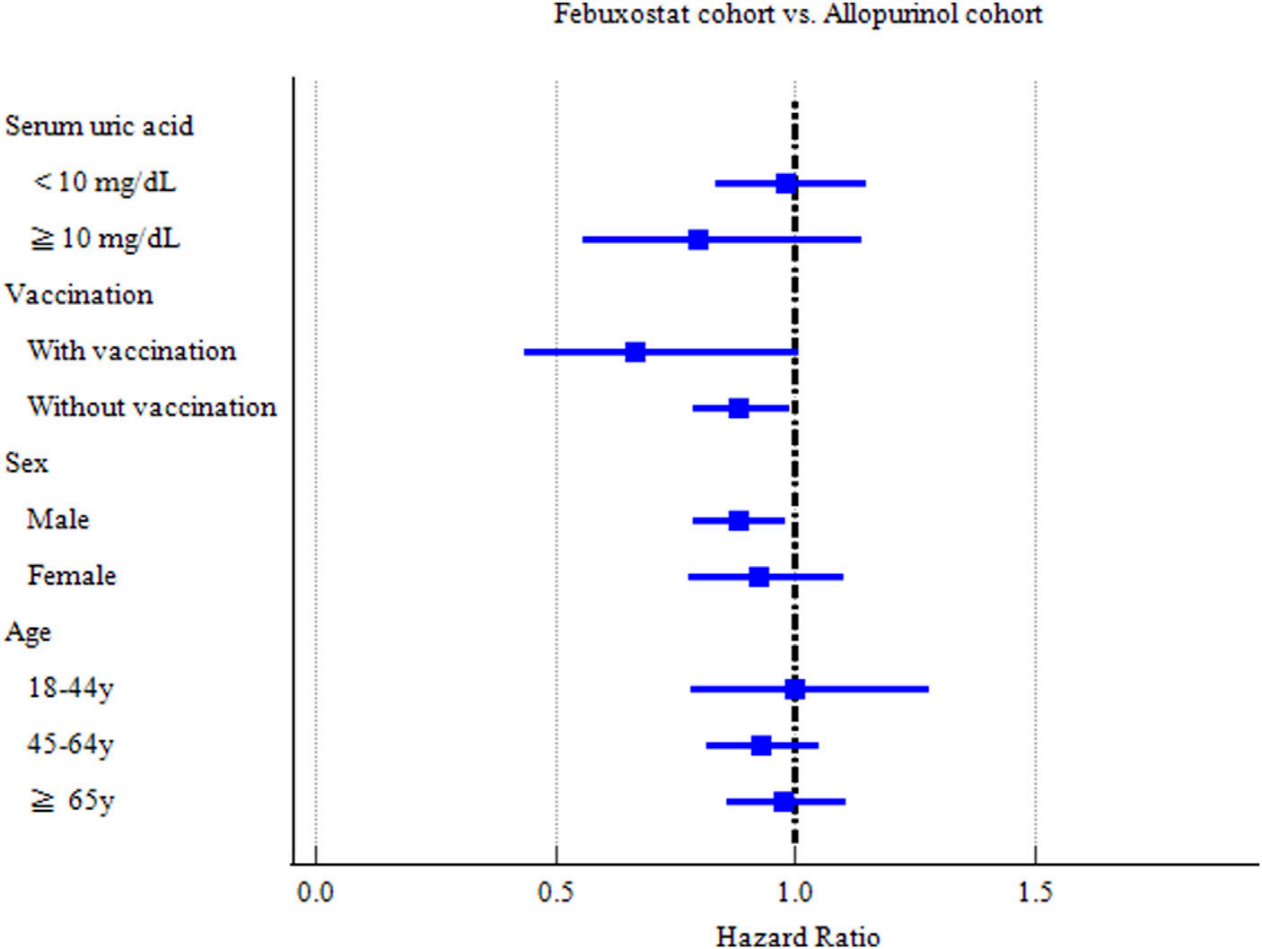
Forest plot of COVID-19 incidence-subgroup analyses.

The risks of CVDs in subgroups were evaluated based on sex, age, vaccination and serum uric acid ( <10 mg/dL or ≧10 mg/dL) (Figure 3).

Hyperuricemia, defined as serum uric acid concentration of 6.8 mg/dL or more, is described as a necessary condition for the development of gout. Gout patients with serum uric acid concentration of more than 10 mg/dL are likely associated with many of the comorbidities. Thus, we conducted subgroup analysis based on serum uric acid level. Febuxostat significantly reduced hospitalization and critical care service among patients with serum uric acid < 10 mg/dL (HR = 0.752 [0.582–0.971]) (HR = 0.710 [0.526–0.959]) respectively (Supplementary Table S3). Febuxostat could reduce the risks of COVID-19 incidence (HR = 0.882 [0.787–0.989] and hospitalization (HR = 0.859[0.741–0.997]) among patients without record of COVID-19 vaccination. It is interesting that Febuxostat could increase the risks of mechanical ventilation among the people vaccinated COVID-19 related vaccines before or up to 7 days after the index date (HR = 3.245 [1.045–10.07]) (Supplementary Table S4).

Male gout patients in the Febuxostat group exhibited a significant reduction in COVID-19 incidence (HR = 0.881 [0.790–0.983]), compared with those in the Allopurinol group (Supplementary Table S5). The middle-aged (aged 45–64 years) (HR = 0.761 [0.633–0.914]) and elderly (aged ≥65 years) (HR = 0.781 [0.669–0.911]) subgroups in the Febuxostat group had a significant reduced risk of hospitalization of COVID-19 patients (Supplementary Table S6).

### Sensitivity analyses

Tophus formation is the cardinal feature of advanced gout (Narang and Dalbeth, 2020; Grubišić and Jordan, 2023). Renal impairment is one of the most common comorbidities of patients with gout (Singh and Gaffo, 2020). To evaluate the risks of outcomes regards to the severity and comorbidities, we performed sensitivity analyses of gout due to renal impairment or with tophus and with different follow up durations.

We found that Febuxostat markedly reduced the hospitalization (HR = 0.652 [0.485–0.877]) in gout patients due to renal impairment or with tophus (Supplementary Table S7). Moreover, Febuxostat significantly reduced the risks of COVID-19 incidence in the follow-up duration of 7 days to 2 years (HR = 0.865 [0.780–0.958]) and 7 days to 3 years (HR = 0.878 [0.801–0.963]). Furthermore, Febuxostat reduced the risks of critical care service (HR = 0.760 [0.580–0.998]) in the follow-up of 7 days–180 days and hospitalization (HR = 0.874[0.772–0.989]) in the follow-up of 7 days to 3 years (Supplementary Table S8). To make the evidence robust, the competing risks of mortality were also evaluated. According to Allopurinol group, Febuxostat significantly reduced the risks of hospitalization (HR = 0.889 [0.791–0.998]) after dealing with the mortality competing risk (Supplementary Table S9).

## Discussion

To the best of our knowledge, this is the first large-scale cohort study to demonstrate that Febuxostat decreases the risk of COVID-19 incidence in gout patients. The present study showed that Febuxostat could significantly reduce the risk of COVID-19 incidence and 3 years follow-up. Furthermore, the consistent findings of significant lower risk of COVID-19 and hospitalization in gout patients were found across four different regression models. We also found that male gout patients in the Febuxostat group exhibited a significant reduction in COVID-19 incidence. In addition, Febuxostat significantly reduced hospitalization and critical care service among patients with serum uric acid < 10 mg/dL. Furthermore, Febuxostat significantly reduced the risk of COVID-19 incidence in the follow-up periods of 7 days to 2 years, and 7 days to 3 years. Moreover, Febuxostat could also reduce the hospitalization among COVID-19 infection in gout patients due to renal impairment or with tophus.

The findings from this study carry substantial clinical implications, particularly in the context of managing gout patients during the COVID-19 pandemic and potentially future viral outbreaks. Given that gout itself is a risk factor for both COVID-19 incidence and severe outcomes, the identification of a commonly used urate-lowering therapy that may also reduce viral infection risk offers a dual therapeutic benefit. Febuxostat’s association with reduced COVID-19 incidence and hospitalization suggests that it may serve as a protective agent beyond its primary indication, especially in high-risk subgroups such as males, the elderly, and those with renal impairment or tophaceous gout.

During the development of gout, monosodium urate crystals are formed around the joints, due to elevated serum uric acid concentrations. As the age increases, men are more likely to suffer from gout than women (Dehlin et al., 2020; Han et al., 2024). In the present study Febuxostat significantly reduced the risks of COVID-19 incidence in male gouts and hospitalization among COVID-19 infection in elder patients (more than 45 years old) compared to the Allopurinol group.

Compared with individuals with a baseline serum urate of <6 mg/dL, the risks of gout in those with a baseline serum urate of ≥10 mg/dL increased to 64 times (Evans et al., 2018). Gout patients with serum uric acid (SUA) concentration of more than 10 mg/dL are likely associated with many of the comorbidities. A Retrospective Study demonstrated that Febuxostat showed efficacy and renal safety in male patients with gout and chronic kidney diseases. Moreover, gout patients with SUA < 10 mg/dL and body weight <70 kg reached more preferrable results (Zhang et al., 2022). In the present study, around 40% of the participants have comorbidities with acute kidney failure and chronic kidney disease. Febuxostat significantly reduced the risks of hospitalization and critical care service in gout patients among COVID-19 infection with SUA <10 mg/dL. Moreover, hospitalization was significantly reduced in gout patients due to renal impairment or with tophus.

COVID-19 vaccination has been demonstrated to be associated with increased odds of gout flare (Lu et al., 2022; He et al., 2024). Similar pathophysiological mechanism of crystal-induced arthritis and the inflammatory response of COVID-19 vaccination might make gout patients more susceptible to vaccine-induced flare than other rheumatic patients (Tai et al., 2022; Perozzo et al., 2022). For gout patients, MSU crystal and prolonged hyperuricemia could increase the inflammatory response to a secondary stimulus such as COVID-19 vaccine (Bodofsky et al., 2020). In the present study, Febuxostat could increase the risk of mechanical ventilation for gout patients with vaccination. In contrast, the risks of COVID-19 incidence and hospitalization were reduced in gout patients without vaccination in the Febuxostat group.

Cytokine storm including inflammatory cytokines IL-1β, IL-6, IL-17, and TNF-α could be provoked in moderate and severe COVID-19 patients (Montazersaheb et al., 2022). From a translational perspective, these results support the reconsideration of Febuxostat as a preferred urate-lowering therapy in gout patients with comorbidities that increase susceptibility to severe viral infections. This is particularly relevant given the drug’s favorable safety profile in patients with renal impairment, a common comorbidity in gout populations. Moreover, the anti-inflammatory properties of Febuxostat, through inhibition of the JAK/STAT and NF-κB pathways and subsequent reduction in pro-inflammatory cytokines such as IL-1β, IL-6, and TNF-α, may underlie its protective effects against COVID-19-related hyperinflammation and organ damage (Hao et al., 2019; Khan et al., 2017; Abo-Youssef et al., 2020). This mechanistic insight not only reinforces the biological plausibility of our findings but also opens avenues for repurposing Febuxostat in other inflammatory or infectious conditions characterized by cytokine dysregulation.

To validate the robustness of our study and reduce the bias, we performed four models of Cox regressions, which all revealed that Febuxostat could reduce the risk of COVID-19 incidence and hospitalization of gout patients infected with COVID-19. Furthermore, after dealing with the mortality competing risk, the results consistent with the former ones.

However, our study has several limitations. First, even though treatment start time of Febuxostat was evaluated, Febuxostat and Allopurinol dose data could not be obtained from the database. We were unable to evaluate dose–response or persistence/time on treatment because the data platform lacks standardized dosing and days-supply/refill information and does not support patient-level time-varying dose construction. As a result, unmeasured differences in titration or persistence between groups could remain and may have influenced effect estimates; findings should be interpreted with this limitation in mind. In addition, there were relatively few Asian people, American Indian or Alaska Native, or Native Hawaiian or Other Pacific Islander participants in our study, which could have caused results to be biased based on race. Collaborative electronic health records was unable to obtain healthcare insurance status during the COVID-19 pandemic, which could confound the results. Second, because index dates were distributed across 2020–2022, many patients had index dates before vaccines became available, resulting in low observed vaccination rates. Additionally, vaccination administered outside of the network may not have been captured, potentially leading to misclassification. Furthermore, our composite outcome definition may have introduced misclassification, as some non-COVID pneumonia cases could have been included, and some infections may have been missed if patients did not seek care or were tested outside the network. Such misclassification is likely non-differential, which would bias effect estimates toward the null. PSM was performed to minimize bias, but misclassification bias and residual confounding could not be completely eliminated due to some disadvantages of an electronic health record database. Moreover, the dataset is not a population-based dataset. Therefore, patients may receive medical treatment at hospitals outside of the collaborative electronic health records platform, resulting in a loss to follow up. Third, a potential limitation of this study is that exposure index dates spanned multiple pandemic waves with evolving testing availability, treatment approaches, and circulating variants. Although the index date distributions overlapped across treatment groups, residual calendar-time confounding cannot be completely excluded. Besides, although we attempted to minimize immortal-time bias by applying a day-7 landmark after the index date and excluding patients who switched between febuxostat and allopurinol during follow-up, residual immortal-time bias cannot be completely ruled out. Readers should interpret the observed associations with caution, recognizing the possibility that any remaining immortal-time bias may have attenuated or inflated the observed effect estimates. Fourth, some combined treatments such as NSAIDs, corticosteroids, and other anti-gout drugs such as colchicine may have effects on the COVID-19 risk of gout patients and some drugs may have complex drug-drug interactions with Febuxostat or Allopurinol. Fifth, we reported exploratory subgroup results for SUA, vaccination record, sex, and age. Because interaction tests were approximated from subgroup HRs rather than estimated within a single model, and no multiplicity control could be implemented, type-I error may be inflated and interaction findings should be interpreted cautiously. Notably, point estimates were directionally consistent (febuxostat associated with lower COVID-19 risk) in SUA >10 mg/dL, vaccinated, female, and ≥65-year strata. Finally, despite propensity score adjustment, residual confounding by indication may persist because febuxostat is often selected for patients with allopurinol intolerance/contraindications or chronic kidney disease, and we could not fully capture gout severity (e.g., tophi, flare frequency, serum urate trajectories), provider preference, or access to care. The primary estimate for COVID-19 infection (HR = 0.878; 95% CI, 0.801–0.963) corresponds to E-values of 1.54 for the point estimate and 1.24 for the upper CI bound, indicating that an unmeasured confounder associated with both treatment assignment and infection risk by a risk ratio of ≥1.54 could fully explain the observed association, whereas a confounder with associations of ∼1.24 could render the result non-significant. Accordingly, our findings should be interpreted with caution in light of potential residual bias.

Future research should include randomized controlled trials to confirm these observational findings and elucidate the optimal dosing and timing of Febuxostat administration relative to vaccination and infection exposure. Additionally, studies exploring the effect of Febuxostat on other respiratory viruses or post-acute sequelae of COVID-19 could further expand its clinical utility.

## Conclusion

Taken together, in this retrospective cohort study, Febuxostat was associated with lower risks of COVID-19 incidence in gout patients.

## Data Availability

The original contributions presented in the study are included in the article/Supplementary Material, further inquiries can be directed to the corresponding authors.
