# Supplementary material for: Febuxostat may decrease the incidence of COVID-19 infection among patients with gout: a retrospective cohort study
Source: Front Pharmacol. 2025 Oct 29;16:1654173. doi: 10.3389/fphar.2025.1654173 (PMC12605445; doi:10.3389/fphar.2025.1654173)
Supplement: Supplementary file 1 [file Table1.docx]

**Supplementary tables**

**Supplementary Table 1** Other covariates about demographic, lifestyles, medical utilization, comorbidities, procedure/medication and laboratory tests

| No | covariates | Code |
| --- | --- | --- |
| 1 | **Demographic**:  Age  Sex |  |
|  | Race |  |
|  | Persons with potential health hazards related to socioeconomic and psychosocial circumstances | ICD10= Z55-Z65 |
| 2 | **Lifestyles**:  Tobacco use (proxy to smoking)  Nicotine dependence (proxy to smoking)  Alcohol related disorders (proxy to alcohol drinking) | ICD10= Z72.0  ICD10= F17  ICD10= F10 |
| 3 | **Medical utilization**:  Office or Other Outpatient Services  Preventive Medicine Services  Emergency Department Services  Hospital Inpatient Services | procedure=1013626  procedure=1013829  procedure=1013711  procedure=1013659 |
| 4 | **Comorbidities**:  Hypertensive diseases  Heart failure  Ischemic heart diseases  Atrial fibrillation and flutter  Nontraumatic intracerebral hemorrhage  Cerebral infarction  Other peripheral vascular diseases  Atherosclerosis  Diabetes mellitus  Overweight and obesity  Disorders of lipoprotein metabolism  Neoplasms  Chronic lower respiratory diseases  Anxiety, stress-related, somatoform and other nonpsychotic mental disorders  Mood [affective] disorders  Unspecified dementia  Depressive episode  Noninfective enteritis and colitis  Diseases of liver  Sleep disorder  Psoriasis  Acute kidney failure and chronic kidney disease  Systemic lupus erythematosus (SLE)  Dermatopolymyositis  Disorders of bone density and structure  Alzheimer's disease  Conjunctivitis | ICD10=I10-16  ICD10= I50  ICD10=I20-I25  ICD10=I48  ICD10=I61  ICD10= I63  ICD10=I73  ICD10=I70  ICD10= E08-E13  ICD10=E66  ICD10=E78  ICD10= C00-D49  ICD10=J40-47  ICD10=F40-48  ICD10=F30-39  ICD10=F03  ICD10=F32  ICD10=K50-K52  ICD10=K70-77  ICD10=G47  ICD10=L40  ICD10= N17- N19  ICD10=M32  ICD10=M33  ICD10=M80-85  ICD10=G30  ICD10= H10 |
| 5 | **Procedure/Medication:**  COVID-19 vaccine  Anti-inflammatory and antirheumatic products, non-steroids  Corticosteroids for systemic use  Diuretics  Ethambutol  Pyrazinamide  **Other antigout**  Colchicine  Probenecid  Pegloticase  lesinurad  sulfinpyrazone | ATC=M01A  ATC=H02  ATC=C03  M=4110  M=8987  M=2683  M=8698  M=1011650  M=1731031  M=10205 |
| 6 | **Laboratory tests:**  Serum uric acid (≥ 10 mg/dL)  BMI (obesity, ≥ 30 kg/m^2^)  serum creatinine (≥1.5mg/dL) | L=9071  L=9083  L=9024 |

**Supplementary Table 2.** Risk of outcomes (adjusted different variables)

| Outcomes  (Febuxostat vs. Allopurinol cohort) | Hazard ratio (95% CI) | | | |
| --- | --- | --- | --- | --- |
|  | Model 1^a^ | Model 2^b^ | Model 3^c^ | Model 4^d^ |
| Disease incidence |  |  |  |  |
| COVID-19 incidence | **0.907 (0.854-0.963)** | **0.882 (0.814-0.957)** | **0.878 (0.810-0.951)** | **0.893 (0.823-0.969)** |
| Medical utilization |  |  |  |  |
| Hospitalization | **0.920 (0.865-0.978)** | 0.975 (0.896-1.060) | 0.953 (0.877-1.036) | 0.950 (0.874-1.034) |
| Critical care service | 0.974 (0.901-1.052) | 1.102 (0.987-1.230) | 1.076 (0.965-1.200) | 0.979 (0.880-1.090) |
| Mechanical ventilation | **1.116 (1.007-1.237)** | **1.246 (1.072-1.448)** | **1.238 (1.066-1.437)** | 1.067 (0.923-1.233) |
| All-cause mortality |  |  |  |  |
| Deceased | **1.188 (1.121-1.260)** | **1.222 (1.123-1.330)** | **1.262 (1.160-1.374)** | **1.131 (1.041-1.229)** |

Note:

CI: Confidence interval; COVID-19: Coronavirus Disease-2019;

a. Crude, before matching.

b. Propensity score matching was performed on age at index, gender, and race.

c. Propensity score matching was performed on age at index, gender, race, socioeconomic status, lifestyles (such as tobacco use, nicotine dependence, and alcohol related disorders), and medical utilization (including preventive medicine services, hospital inpatient services, emergency department services, office or other outpatient services, and COVID-19 vaccination).

d. Propensity score matching was performed on all listed characteristics.

* Proportionality <0.001

**SupplementaryTable 3.** Risk of outcomes _stratified by serum uric acid

| Outcomes  (Febuxostat vs. Allopurinol cohort) | Adjusted ^a^ Hazard ratio (95% CI) | | p for interaction^d^ |
| --- | --- | --- | --- |
|  | SUA < 10 mg/dL^b^  (n=2202) | SUA ≧ 10 mg/dL^c^  (n=593) |  |
| Disease incidence |  |  |  |
| COVID-19 incidence | 1.001 (0.868-1.153) | **0.709 (0.540-0.930)** | **0.027** |
| Medical utilization |  |  |  |
| Hospitalization | 0.938 (0.782-1.125) | 0.896 (0.610-1.317) | 0.832 |
| Critical care service | **0.792 (0.630-0.995)** | **1.488 (1.033-2.143)** | **0.004** |
| Mechanical ventilation | 0.892 (0.668-1.191) | 1.247 (0.701-2.216) | 0.307 |
| All-cause mortality |  |  |  |
| Deceased | 1.012 (0.857-1.195) | 1.068 (0.821-1.389) | 0.734 |

Note:

CI: Confidence interval; SUA: serum uric acid; COVID-19: Coronavirus Disease-2019;

1. Propensity score matching was performed on all listed characteristics.
2. SUA <10 mg/dl and never ≥ 10 mg/dl within 6 months before or up to 1 week after the index date;
3. SUA ≥ 10 mg/dl within 6 months before or up to 1 week after the index date;
4. P for interaction values were approximated from subgroup-specific HRs and CIs (normal approximation; assumes independence)

**SupplementaryTable 4.** Risk of outcomes _stratified by vaccination

| Outcomes  (Febuxostat vs. Allopurinol cohort) | Adjusted ^a^ Hazard ratio (95% CI) | | p for interaction^d^ |
| --- | --- | --- | --- |
|  | With vaccination^b^  (n=305) | Without vaccination^c^  (n=6167) |  |
| Disease incidence |  |  |  |
| COVID-19 incidence | **0.567 (0.359-0.896)** | 0.915 (0.829-1.009) | **0.044** |
| Medical utilization |  |  |  |
| Hospitalization | 1.250 (0.711-2.195) | **0.847 (0.772-0.930)** | 0.181 |
| Critical care service | 0.853 (0.455-1.602) | 0.915 (0.811-1.032) | 0.830 |
| Mechanical ventilation | 1.137 (0.412-3.135) | 1.087 (0.927-1.275) | 0.931 |
| All-cause mortality |  |  |  |
| Deceased | 0.840 (0.424-1.668) | 1.081 (0.992-1.179) | 0.473 |

Note:

CI: Confidence interval; COVID-19: Coronavirus Disease-2019;

1. Propensity score matching was performed on all listed characteristics.
2. Vaccinated COVID-19 related vaccines before or up to 7 days after index date;
3. No record of vaccination ever documented in their electronic medical records.
4. P for interaction values were approximated from subgroup-specific HRs and CIs (normal approximation; assumes independence)

**SupplementaryTable 5.** Risk of outcomes _stratified by sex

| Outcomes  (Febuxostat vs. Allopurinol cohort) | Adjusted ^a^ Hazard ratio (95% CI) | | p for interaction^b^ |
| --- | --- | --- | --- |
|  | Male  (n=5678) | Female  (n=2129) |  |
| Disease incidence |  |  |  |
| COVID-19 incidence | 0.958 (0.870-1.054) | **0.817 (0.699-0.956)** | 0.089 |
| Medical utilization |  |  |  |
| Hospitalization | 0.968 (0.876-1.069) | 0.891 (0.760-1.044) | 0.385 |
| Critical care service | 0.967 (0.851-1.099) | 0.858 (0.710-1.036) | 0.304 |
| Mechanical ventilation | 1.045 (0.878-1.245) | 1.098 (0.847-1.423) | 0.756 |
| All-cause mortality |  |  |  |
| Deceased | 1.097 (0.992-1.213) | 1.151 (0.994-1.333) | 0.596 |

Note:

CI: Confidence interval; COVID-19: Coronavirus Disease-2019;

1. Propensity score matching was performed on all listed characteristics.
2. P for interaction values were approximated from subgroup-specific HRs and CIs (normal approximation; assumes independence)

**SupplementaryTable 6.** Risk of outcomes _stratified by age

| Outcomes  (Febuxostat vs. Allopurinol cohort) | Adjusted ^a^ Hazard ratio (95% CI) | | | p for interaction^c^ | | |
| --- | --- | --- | --- | --- | --- | --- |
|  | 18-44y  (n=964) | 45-64y  (n=3699) | ≧ 65y  (n=4474) | 18-44y  vs.  45-64y | 45-64y  vs.  ≧ 65y | 18-44y  vs.  ≧ 65y |
| Disease incidence |  |  |  |  |  |  |
| COVID-19 incidence | 0.957 (0.773-1.184) | 0.946 (0.847-1.057) | **0.824 (0.739-0.918)** | 0.924 | 0.080 | 0.220 |
| Medical utilization |  |  |  |  |  |  |
| Hospitalization | 1.222 (0.899-1.660) | 0.980 (0.859-1.118) | 0.915 (0.824-1.016) | 0.194 | 0.424 | 0.080 |
| Critical care service | 1.033 (0.685-1.557) | 0.991 (0.839-1.171) | **0.827 (0.729-0.938)** | 0.854 | 0.089 | 0.310 |
| Mechanical ventilation | 0.963 (0.550-1.686) | 1.041 (0.834-1.301) | 0.995 (0.838-1.180) | 0.800 | 0.752 | 0.912 |
| All-cause mortality |  |  |  |  |  |  |
| Deceased | 1.039 (0.698-1.547) | **1.165 (1.000-1.357)^b^** | 1.080 (0.985-1.185) | 0.598 | 0.405 | 0.852 |

Note:

CI: Confidence interval; COVID-19: Coronavirus Disease-2019;

1. Propensity score matching was performed on all listed characteristics.
2. Log-Rank test, p=0.049.
3. P for interaction values were approximated from subgroup-specific HRs and CIs (normal approximation; assumes independence)

**Supplementary Table 7.** Risk of outcomes_among severe patient (Gout due to renal impairment (M10.3) or M1A.XXX1 with tophus)

| Outcomes | Patients with outcome | | Adjusted ^a^ hazard ratio (95% CI) |
| --- | --- | --- | --- |
|  | Febuxostat cohort  (n=1082) | Allopurinol cohort  (n=1082) |  |
| Disease incidence |  |  |  |
| COVID-19 incidence | 198 | 214 | 0.937 (0.773-1.137) |
| Medical utilization |  |  |  |
| Hospitalization | 72 | 112 | **0.652 (0.485-0.877)** |
| Critical care service | 83 | 88 | 0.933 (0.691-1.259) |
| Mechanical ventilation | 34 | 44 | 0.781 (0.499-1.222) |

Note:

CI: Confidence interval.

1. Propensity score matching was performed on all listed characteristics.

**Supplementary Table 8.** Risk of outcomes (different follow up duration)

| Outcomes  (Febuxostat vs. Allopurinol) | Adjusted^a^ hazard ratio (95% CI) | | | | |
| --- | --- | --- | --- | --- | --- |
|  | 7 days to 90 days | 7 days to 180 days | 7 days to 1y | 7 days to 2y | 7 days to 3y |
| Disease incidence |  |  |  |  |  |
| COVID-19 incidence | 0.810 (0.597-1.099) | 0.820 (0.668-1.006) | 0.895 (0.779-1.028) | **0.865 (0.780-0.958)** | **0.878 (0.801-0.963)** |
| Medical utilization |  |  |  |  |  |
| Hospitalization | 0.894 (0.688-1.162) | 0.846 (0.691-1.036) | 0.895 (0.765-1.046) | 0.902 (0.789-1.030) | **0.874 (0.772-0.989)** |
| Critical care service | 0.869 (0.619-1.218) | **0.760 (0.580-0.998)** | 0.883 (0.721-1.082) | 0.867 (0.734-1.023) | 0.880 (0.755-1.025) |
| Mechanical ventilation | 0.872 (0.554-1.372) | 0.851 (0.590-1.228) | 0.982 (0.748-1.289) | 0.998 (0.794-1.254) | 0.965 (0.782-1.192) |

Note:

CI: Confidence interval.

1. Propensity score matching was performed on all listed characteristics.

**Supplementary Table 9.** Risk of outcomes (deal with competing risk)

| Outcomes | Patients with outcome | | Adjusted ^a^ hazard ratio (95% CI) |
| --- | --- | --- | --- |
|  | Febuxostat cohort  (n=5462) | Allopurinol cohort  (n=5462) |  |
| Disease incidence |  |  |  |
| COVID-19 incidence | 1317 | 1405 | 0.943 (0.874-1.016) |
| Medical utilization |  |  |  |
| Hospitalization | 541 | 597 | **0.889 (0.791-0.998)** |
| Critical care service | 611 | 585 | 1.051 (0.939-1.178) |
| Mechanical ventilation | 587 | 543 | 1.097 (0.976-1.232) |

Note:

CI: Confidence interval.

1. Propensity score matching was performed on all listed characteristics.
